# Dielectrophoresis-Based Selective Droplet Extraction Microfluidic Device for Single-Cell Analysis

**DOI:** 10.3390/mi14030706

**Published:** 2023-03-22

**Authors:** Seito Shijo, Daiki Tanaka, Tetsushi Sekiguchi, Jun-ichi Ishihara, Hiroki Takahashi, Masashi Kobayashi, Shuichi Shoji

**Affiliations:** 1 Major in Nanoscience and Nanoengineering, Waseda University, 3-4-1 Okubo, Shinjuku, Tokyo 145-0065, Japan; maajiired@gmail.com (M.K.);; 2 Research Organization for Nano & Life Innovation, Waseda University, 513 Tsurumakicho, Shinjuku, Tokyo 162-0041, Japan; 3 Medical Mycology Research Center, Chiba University, 181 Inohana, Chuo, Chiba 260-8673, Japan; 4 Molecular Chirality Research Center, Chiba University, 1-33 Yayoi-cho, Inage-ku, Chiba 263-8522, Japan; 5 Plant Molecular Science Center, Chiba University, 181 Inohana, Chuo, Chiba 260-8673, Japan

**Keywords:** microfluidics, microdroplet, dielectrophoresis (DEP), selective extraction

## Abstract

We developed a microfluidic device that enables selective droplet extraction from multiple droplet-trapping pockets based on dielectrophoresis. The device consists of a main microchannel, five droplet-trapping pockets with side channels, and drive electrode pairs appropriately located around the trapping pockets. Agarose droplets capable of encapsulating biological samples were successfully trapped in the trapping pockets due to the difference in flow resistance between the main and side channels. Target droplets were selectively extracted from the pockets by the dielectrophoretic force generated between the electrodes under an applied voltage of 500 V. During their extraction from the trapping pockets, the droplets and their contents were exposed to an electric field for 400–800 ms. To evaluate whether the applied voltage could potentially damage the biological samples, the growth rates of *Escherichia coli* cells in the droplets, with and without a voltage applied, were compared. No significant difference in the growth rate was observed. The developed device enables the screening of encapsulated single cells and the selective extraction of target droplets.

## 1. Introduction

Cell-array-based microfluidic devices are powerful tools for single-cell analysis since they can be used to isolate individual cells from a cell population for prolonged observation. By trapping cells in the flow path, various aspects of cell behavior can be examined, including cell interactions [1], drug responses [2,3], and protein expression [4,5]. Several approaches, such as those based on gravity [2,3,4,6], hydrodynamics [1,7,8], optical tweezers [9], and dielectrophoresis (DEP) [10,11], have been applied to microfluidic devices to capture single cells. In addition, many studies require selective extraction of the cells from the device after screening for subsequent off-chip analysis of more specific responses. Recently, Lv et al. [12] and Zhu et al. [13] successfully trapped cells hydrodynamically and selectively extracted them from the device by DEP, while Kim et al. [14] were able to trap cells using microvalves and selectively extract them to specific locations by applying backflow. However, conventional microfluidic devices capture bare cells directly in the channel, which can cause cell damage due to the pressure used for capture or contamination in the fluid. In addition, an analysis of samples on the single-micrometer scale, such as *Escherichia coli*, requires correspondingly smaller microchannels, for which it is more difficult to precisely fabricate extraction mechanisms such as valves and electrodes within the channels.

To overcome these difficulties, microdroplets can be used to protect cells from pressure and contamination, and the droplet size can be adjusted for easier handling. In addition, microdroplets can be trapped in the channel in the same manner as cell arrays, and several studies have reported screening by arraying droplets [15,16,17]. However, an effective platform for selectively extracting only the target droplets from the arrayed droplets after screening has not yet been developed. Tan and Takeuchi [18] succeeded in selectively extracting cell-encapsulating alginate droplets using microbubbles generated by a laser, though this process requires a complex experimental apparatus and the heat from the laser may damage the cells in the droplets. Toprakcioglu and Knowles [19] accomplished the sequential release of microdroplets without damaging cells by creating backflow in the channel after trapping the droplets, although this approach does not allow for selective extraction of the target droplets. Sorting devices for the selective extraction of droplets have also been developed [20,21,22,23,24], however, devices without trap sections for droplets make it difficult to observe droplets over time, and continuous observation of the same droplets requires the addition of labeling factors to the droplets or cells. Thus, there is a need for devices with simple structures that can selectively extract droplets without causing significant damage to the sample inside the droplets.

In this paper, we propose a simple new device for the trapping and selective extraction of droplets, in which dielectrophoresis (DEP) is employed as the operating principle for selectively extracting the droplets. Agresti et al. [22], Schütz et al. [23], and Isozaki et al. [24] have successfully performed a high-throughput sorting of droplets using DEP. Furthermore, Jiang et al. [25] reported on the successful sorting and separation of droplets by DEP and its ease of control. Those studies demonstrate that precise manipulation of droplets can be achieved with a simple structure, such as one with electrodes placed around the flow channel. Using the proposed device with a simpler structure than conventional devices, a target droplet can be extracted from an array of droplets. The functionality of the device was verified using *E. coli*-encapsulating agarose droplets.

## 2. Materials and Methods

### 2.1. Operating Principle

**Figure 1:**
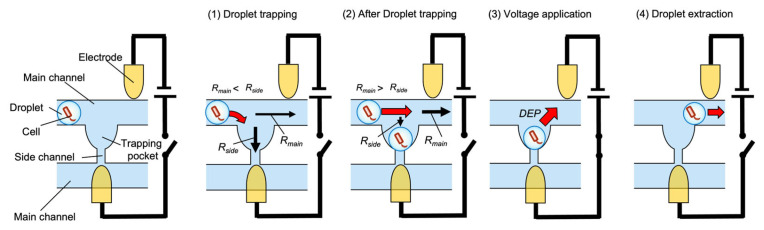
Operating principle of the microdroplet manipulation system.

The operating principle of the microdroplet manipulation system is depicted in Figure 1. We considered a simple system that uses hydrodynamics and DEP to selectively extract droplets.

A hydrodynamic mechanism is employed to trap microdroplets in the channel. Microdroplets are guided into the trapping pockets by making the flow resistance of the pocket and side channel (*R*_side_) smaller than that of the main channel (*R*_main_). When a droplet is trapped in a pocket, the flow resistance increases and subsequent droplets pass through the main channel to the next pocket without being trapped in the same pocket section [12,13,18].

DEP is used to extract a microdroplet from a pocket. Microparticles such as microdroplets become polarized when an external electric field is applied, forming electric dipoles. If the applied electric field is nonuniform, different electric fields will be generated at the ends of the microparticle and the microparticle experiences a dielectrophoretic force. The dielectrophoretic force acting on a microparticle can be expressed by the following equation:

(1) $$F_{\mathrm{DEP}}=2\pi \varepsilon_{m}r^{3}Re\left(f_{\mathrm{CM}}\right)\nabla |E{|}^{2},$$

where *ε*_m_ is the permittivity of the surrounding liquid medium, *r* is the particle radius, *Re* indicates the real part of a complex function, *f*_CM_ is the Clausius–Mossotti factor, and ∇|*E*|^2^ is the gradient of the square of the electric field. Here, *f*_CM_ is given by

(2) $$f_{\mathrm{CM}}=\frac{\varepsilon_{p}^{*}-\varepsilon_{m}^{*}}{\varepsilon_{p}^{*}+2\varepsilon_{m}^{*}},$$

where $\varepsilon_{p}^{*}$ is the complex permittivity of the microparticle and $\varepsilon_{m}^{*}$ is the complex permittivity of the surrounding liquid medium. When *f*_CM_ > 0, the microparticle is attracted toward the stronger electric field, which is referred to as positive dielectrophoresis (P-DEP); conversely, when *f*_CM_ < 0, the microparticle is attracted toward the weaker electric field, which is referred to as negative dielectrophoresis (N-DEP). When a DC electric field is applied [26], *f*_CM_ is given by the following equation:

(3) $$f_{\mathrm{CM}}=\frac{\sigma_{p}-\sigma_{m}}{\sigma_{p}+2\sigma_{m}},$$

where *σ*_p_ and *σ*_m_ are the electrical conductivities of the microparticle and surrounding liquid medium, respectively. In other words, the electrical conductivity of the materials determines the direction of the dielectrophoretic force. If the electrical conductivity of the microparticle is higher than that of the surrounding liquid medium, P-DEP occurs, and if the electrical conductivity of the microparticle is lower than that of the surrounding liquid medium, N-DEP occurs. In this work, *σ*_p_ > *σ*_m_ because an agarose solution (microdroplets) was used as the microparticles, and mineral oil, which is an electrical insulator, was used as the surrounding liquid medium (carrier). Therefore, the microparticles in the microchannel would be expected to undergo P-DEP. The device should be designed to concentrate the electric field outside of the trapping pockets.

### 2.2. Device Design and Fabrication Process

**Figure 2:**
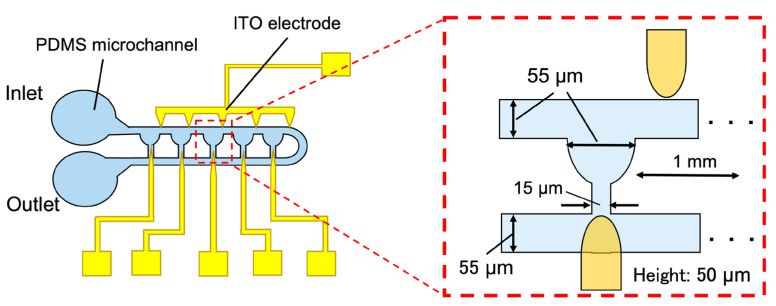
Schematic view and dimensions of the device.

Figure 2 presents the device design and dimensions. The device consisted of a main channel, five droplet-trapping pockets with side channels, and electrodes on a glass substrate. As described in Section 2.1, the device was designed such that droplets would be trapped in the pockets due to the difference in flow resistance between the main channel and the pocket structure. The upper electrodes were placed on the right side of the pockets to prevent extracted droplets from being trapped back into the pocket.

The channel height was 50 µm and the channel width and pocket diameter were 55 µm. Side channels were designed with 1 mm spacing and a width of 15 µm.

The microchannel was fabricated by soft lithography. A mold was made with an SU-8 photoresist (SU-8 3025). Polydimethylsiloxane (PDMS) and a curing agent were mixed in a ratio of 10:1 (*w*/*w*) and poured into the mold, thermally cured, and then removed from the mold to form the microchannel. Indium tin oxide (ITO) electrodes were fabricated by lithography and etching processes. The electrode design was patterned using AZ resist (AZ P4620) on a glass substrate coated with 700-nm-thick ITO, followed by wet etching of the excess ITO using 15% hydrochloric acid and removal of the resist using acetone and 2-propanol. Finally, the microchannel and ITO electrode on the substrate were aligned and bonded under a microscope (IX71, Olympus, Tokyo, Japan) after a plasma treatment (Aiplasma, Matsushita Electric Works, Forest Grove City, OR, USA). The bonded devices were then baked at 100 °C for 30 min to improve the bonding strength.

### 2.3. Reagent Preparation

Agarose solution was used for the microdroplets. Agarose and ultrapure water were mixed in a ratio of 1:99 (*w*/*w*) to obtain an agarose solution with a concentration of 1 wt%. Mineral oil was used as the carrier oil. Span 80 was added to the mineral oil at a concentration of 1 wt% as a surfactant.

### 2.4. Cell Preparation

*E. coli* cells were used as a model biological sample. The *E. coli* strain (DH 10B) used in this study harbors a transcriptional fusion of a promoter-less gfp gene fused to a trc promoter. We cultured the cells in Luria–Bertani (LB) broth (Difco) supplemented with 0.1 mM isopropyl-β-d-thiogalactopyranoside at 37 °C with shaking. To prepare *E. coli* cells for enclosure within the droplets, an aliquot (2 µL) of the glycerol stock of *E. coli* cells was inoculated into an LB medium (2 mL) and incubated at 37 °C for 15 h with shaking. The cell culture was diluted to an OD_600_ value of 0.03 with liquefied agarose gel.

### 2.5. Agarose Droplet Preparation

**Figure 3:**
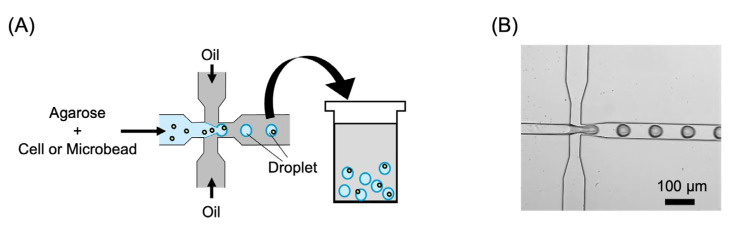
Droplet generation using the microfluidic device. (**A**) Schematic view. (**B**) Microscope image.

Agarose droplets encapsulating *E. coli* or fluorescent microbeads were prepared shortly before their introduction into the microfluidic device. Agarose solutions containing *E. coli* or fluorescent microbeads and mineral oil were introduced into a microfluidic device for droplet generation. Figure 3 shows a schematic view and microscope image of droplet generation using the microfluidic device. The droplets were then collected and allowed to stand at 4 °C for 1 h to allow them to gel completely.

### 2.6. Experimental Setup

The agarose gel droplets and mineral oil were introduced into the device via syringes (1750 CX, Hamilton, Reno, NV, USA) driven by syringe pumps (Legato 100, KD Scientific, Holliston, MA, USA). A DC voltage/current source (6166, ADCMT) was used to apply a voltage between the electrode pairs. In this case, the anode and cathode were connected to the upstream and downstream electrodes, respectively. The experimental results were observed using a microscope (IX71, Olympus, Tokyo, Japan) and a high-speed camera (FASTCAM Mini AX, Photron, Tokyo, Japan).

## 3. Results and Discussion

### 3.1. Finite Element Simulations

Accurate droplet extraction requires an optimized electric field distribution. Finite element simulations were performed using COMSOL Multiphysics 5.4 to investigate the electric field distribution in the device.

**Figure 4:**
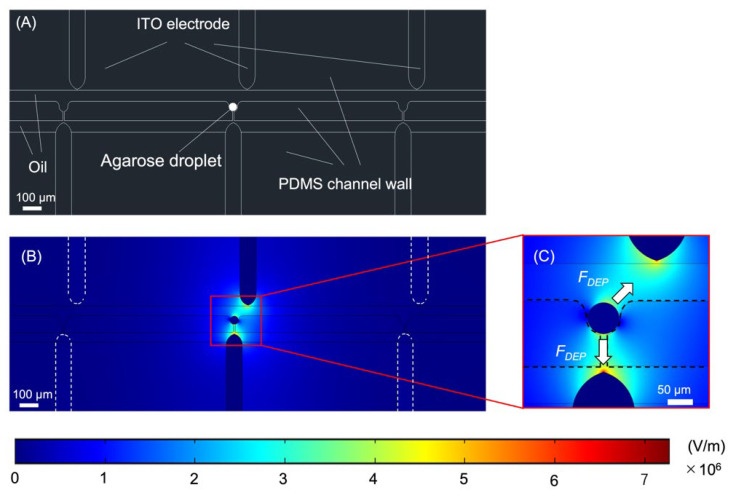
Simulation results for the electric field distribution. (**A**) Schematic view of the model used for the simulation. (**B**) Electric field distribution throughout the model. (**C**) Electric field distribution around the central trapping pocket and the direction of *F*_DEP_ generation.

A model featuring three pockets and three electrode pairs was used for the simulations. The relative permittivity was set to 78 for the microdroplet (Agarose droplet), 2.5 for the liquid medium surrounding the droplet (Oil), 2.75 for the channel wall (PDMS channel wall), and 1 for the electrode (ITO). The distribution of the electric field gradient in the channel was simulated with the electrostatics interface of the AC/DC module, and a DC voltage of 500 V was applied to the electrode pair at the center of the model. The mesh used was a physics-controlled mesh and the size was finer. Figure 4 presents the simulation results at time t, right when the voltage is applied. Only one electrode pair was activated at a time; consequently, the other trapping pockets were not exposed to the electric field (Figure 4B). This result indicated that DEP should occur only for the corresponding droplets when a voltage is applied. In addition, the electric field was strongest at the tip of the electrode pair through which the voltage was applied. Therefore, the dielectrophoretic force (*F*_DEP_) experienced by the droplet can be considered to occur in the direction shown in Figure 4C. Since the *F*_DEP_ generated in the direction of the lower electrode is blocked by the trapping structure, the droplet can be expected to move in the direction of the upper electrode. The simulation results also indicate that droplets are exposed to an electric field, and there is a possibility that the cells within the droplets may be affected by the voltage. Several studies have reported that applied DEP can cause stress to cells [27,28]. Desai et al. [29] investigated the effects of DEP on NIH3T3 fibroblasts and reported increased stress on the cells with increasing field voltage and exposure time. Therefore, this study should investigate the effects of the extraction process on the cells within the droplet.

### 3.2. Droplet Trapping and Extraction

**Figure 5:**
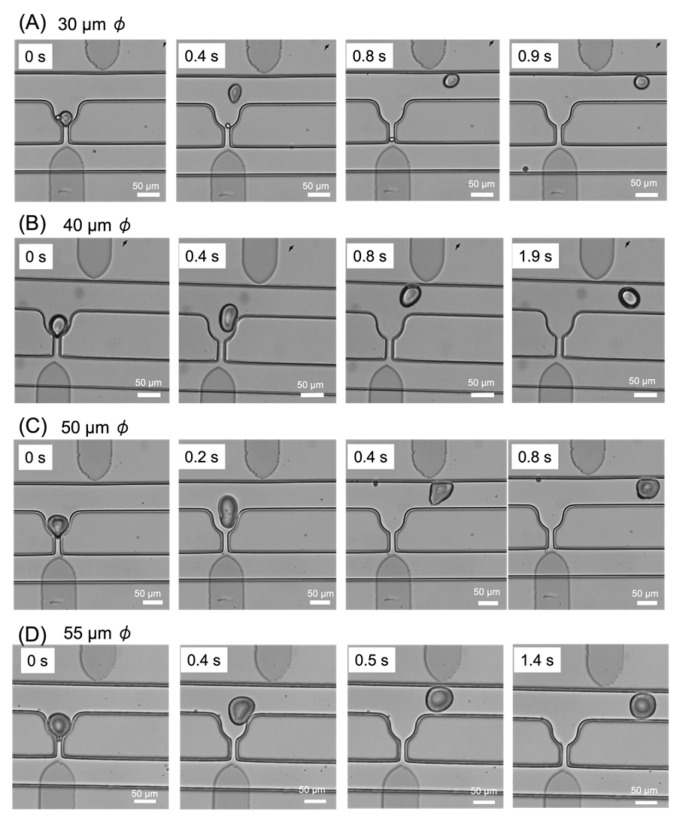
Images of droplet trapping and extraction. (**A**) 30 μm, (**B**) 40 μm, (**C**) 50 μm, and (**D**) 55 μm.

In this subsection, we discuss droplet trapping and extraction, which are the key aspects of this study. Agarose droplets and mineral oil were injected from the inlet at a flow rate of 0.2 μL/min. Figure 5 (Supplementary Materials, Video S1) shows the trapping and extraction of four droplets of different diameters ranging from 30 to 55 μm. After the trapping of the droplets, a voltage was applied to the electrodes to generate P-DEP between them. The voltage was applied continuously until the droplets had been extracted. The application of a voltage of 500 V to the electrodes for 400–800 ms caused the trapped droplets of all sizes (30–55 μm) to be extracted from the pocket as they were pulled toward the upper electrode. The results indicate that the generated dielectrophoretic force was sufficient to extract the droplets. Although successful extraction was confirmed at all voltages ranging from 500 V to 1 kV, the lowest applied voltage of 500 V was selected as the optimal value for cell experiments. A released droplet may become trapped again in an open trapping pocket further downstream. When this occurs, it is necessary to re-extract the droplet by applying a voltage to the electrode of the downstream pocket. These experimental results demonstrate that the fabricated device was capable of trapping and extracting droplets of various sizes.

### 3.3. Selective Droplet Extraction

**Figure 6:**
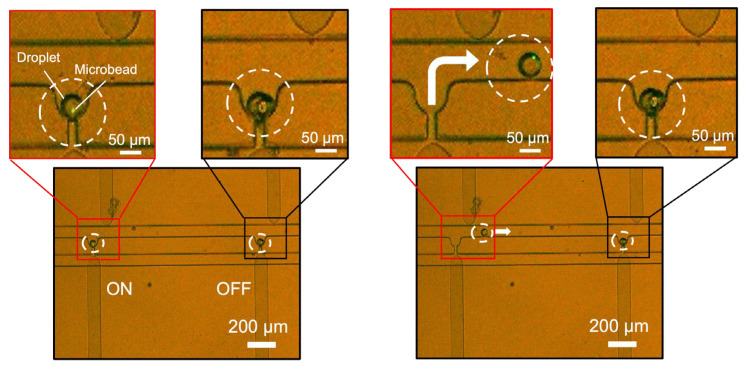
Images of the selective extraction of target droplets. Voltage was applied to the left electrode pair only.

As a next step, we observed two pockets simultaneously to evaluate the capability of the device for selective droplet extraction. For this experiment, we used agarose droplets encapsulating fluorescent microbeads (3 μm diameter). Figure 6 (Supplementary Materials, Video S2) shows the obtained images. Agarose droplets encapsulating fluorescent microbeads and mineral oil were injected from the inlet at a flow rate of 0.2 μL/min to trap the droplets in the pockets. The trapped droplet on the left of the image contained a microbead, whereas the droplet on the right was empty. When 500 V were applied only to the pair of electrodes corresponding to the left pocket, only the droplet on the left side was extracted. These results suggest that P-DEP only occurred in the pockets between electrode pairs to which the voltage was applied, thus confirming the feasibility of selective droplet extraction.

### 3.4. Application of the Device to Biological Samples

**Figure 7:**
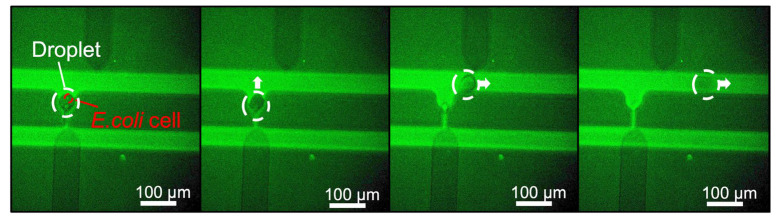
Trapping and extraction of agarose droplets encapsulating *E. coli*.

The ability to conduct reproducible experiments using cells was next examined as an important element for validating the biological utility of the device. Figure 7 (Supplementary Materials, Video S3) shows the trapping and extraction of agarose droplets encapsulating *E. coli* cells. Agarose droplets encapsulating *E. coli* cells and mineral oil were injected at a flow rate of 0.2 µL/min from the inlet and the droplets became trapped in the pockets. Upon applying a voltage of 500 V to the corresponding electrode pair, sufficient P-DEP was generated between the electrodes to extract the droplets. The results indicate that the fabricated device is capable of trapping and extracting droplets encapsulating bacteria and other biomolecules.

### 3.5. Evaluation of Damage to Biological Samples 

**Figure 8:**
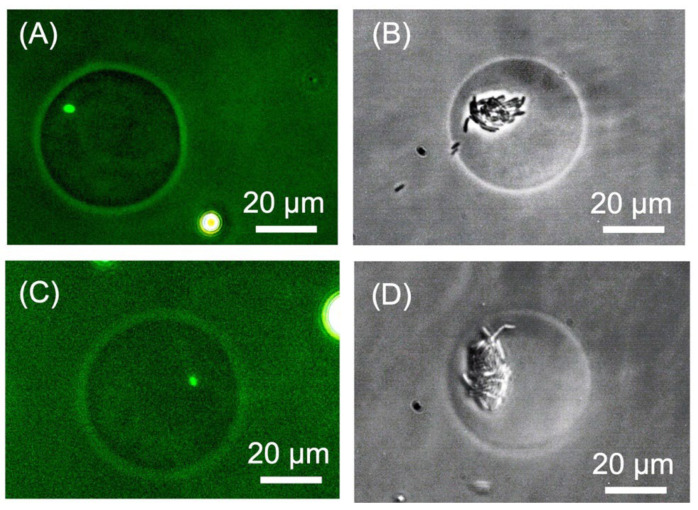
Photomicrographs of agarose droplets encapsulating *E. coli* cells. (**A**) Control droplet after 0 h of incubation, (**B**) Control droplet after 15 h of incubation, (**C**) DEP droplet after 0 h of incubation, and (**D**) DEP droplet after 15 h of incubation.

To evaluate whether the extraction process damaged the *E. coli* cells in the droplets, the specific growth rates of the cells were compared between the droplets subject to an applied voltage (DEP droplets) and those not subject to an applied voltage (control droplets). The droplets were washed to remove oil and incubated in an LB medium at 20 °C. After a period of time, the number of *E. coli* cells in the droplets was counted under a microscope. The number of *E. coli* cells encapsulated in a droplet is determined stochastically according to a Poisson distribution, such that the number of cells in each droplet may vary [30]. Therefore, 15 droplets were randomly selected from the droplet population and the average number of cells was calculated. Table 1 shows the number of *E. coli* cells in the droplets immediately after washing and after 15 h of incubation. In addition, Figure 8 presents images of the DEP and control droplets. The number of *E. coli* cells in the control droplets increased from 0.9 before incubation to 17.8 after 15 h, while the number of *E. coli* cells in the DEP droplets increased from 1.3 before incubation to 21.8 after 15 h. Under constant culture conditions, the specific growth rate of bacteria can be expressed by the following formula [31]:

(4) $$\alpha t=\ln\frac{n}{n_{0}},$$

where α is the specific growth rate, *t* is the incubation time, *n* is the number of bacteria at time *t*, and $n_{0}$ is the initial number of bacteria. The growth rate per hour was calculated to be 0.2 for the control droplets and 0.19 for the DEP droplets. The experiment was repeated three times to confirm the accuracy of the results. The growth rates for the second trial were as follows: control droplets were 0.21 and DEP droplets were 0.21. Similarly, the growth rates for the third trial were: control droplets were 0.22 and DEP droplets were 0.19. The standard deviation of the growth rate was calculated to be 0.006 for control droplets and 0.015 for DEP droplets. Additionally, the standard deviation of the growth rate per hour of the *E. coli* cells in the DEP droplets and the control droplets was calculated to be 0.01. These results indicate that the extraction process used in this study did not have a significant effect on the viability of *E. coli*.

## 4. Conclusions

In this study, we developed a simple new microfluidic device for droplet screening and low-damage selective extraction. For the purposes of the current demonstration, we fabricated a device containing five droplet-trapping pockets with electrode pairs positioned appropriately above and below each trapping pocket to generate a dielectrophoretic force. Experiments involving agarose droplets demonstrated that the device was capable of droplet trapping and selective extraction. Comparative experiments with droplets encapsulating *E. coli* cells confirmed that the extraction process had no significant effect on cell viability. Therefore, we believe that the results of this study will contribute to further progress in droplet-based single-cell analysis.

## Figures and Tables

**Table 1:** Number of *E. coli* cells in agarose droplets.

Trial	Incubation Time (h)	Control Droplets	DEP Droplets
1st	0	0.9	1.3
	15	17.8	21.8
2nd	0	0.8	0.9
	15	18.4	25.6
3rd	0	1.0	1.1
	15	23.2	23.9

## Data Availability

The data presented in this study are available in (Supplementary Materials: Videos S1–S3).

## Supplementary Materials

The following supporting information can be downloaded at: https://www.mdpi.com/article/10.3390/mi14030706/s1: Video S1: droplet trapping and extraction. Video S2: selective extraction of target droplets. Video S3: trapping and extraction of agarose droplets encapsulating *E. coli*.
